# Going the Extra (Synaptic) Mile: Excitotoxicity as the Road Toward Neurodegenerative Diseases

**DOI:** 10.3389/fncel.2020.00090

**Published:** 2020-04-24

**Authors:** Adam Armada-Moreira, Joana I. Gomes, Carolina Campos Pina, Oksana K. Savchak, Joana Gonçalves-Ribeiro, Nádia Rei, Sara Pinto, Tatiana P. Morais, Robertta Silva Martins, Filipa F. Ribeiro, Ana M. Sebastião, Vincenzo Crunelli, Sandra H. Vaz

**Affiliations:** ^1^Instituto de Farmacologia e Neurociências, Faculdade de Medicina da Universidade de Lisboa, Lisbon, Portugal; ^2^Instituto de Medicina Molecular João Lobo Antunes, Faculdade de Medicina da Universidade de Lisboa, Lisbon, Portugal; ^3^Interdisciplinary Nanoscience Center (iNANO), Aarhus University, Aarhus, Denmark; ^4^Neuroscience Division, School of Bioscience, Cardiff University, Cardiff, United Kingdom; ^5^Laboratório de Neurofarmacologia, Instituto Biomédico, Universidade Federal Fluminense, Niterói, Brazil; ^6^Department of Physiology and Biochemistry, Faculty of Medicine and Surgery, University of Malta, Msida, Malta

**Keywords:** excitotoxicity, astrocytes, NMDA receptors, calcium signaling, neurodegenerative diseases, oxidative stress

## Abstract

Excitotoxicity is a phenomenon that describes the toxic actions of excitatory neurotransmitters, primarily glutamate, where the exacerbated or prolonged activation of glutamate receptors starts a cascade of neurotoxicity that ultimately leads to the loss of neuronal function and cell death. In this process, the shift between normal physiological function and excitotoxicity is largely controlled by astrocytes since they can control the levels of glutamate on the synaptic cleft. This control is achieved through glutamate clearance from the synaptic cleft and its underlying recycling through the glutamate-glutamine cycle. The molecular mechanism that triggers excitotoxicity involves alterations in glutamate and calcium metabolism, dysfunction of glutamate transporters, and malfunction of glutamate receptors, particularly N-methyl-D-aspartic acid receptors (NMDAR). On the other hand, excitotoxicity can be regarded as a consequence of other cellular phenomena, such as mitochondrial dysfunction, physical neuronal damage, and oxidative stress. Regardless, it is known that the excessive activation of NMDAR results in the sustained influx of calcium into neurons and leads to several deleterious consequences, including mitochondrial dysfunction, reactive oxygen species (ROS) overproduction, impairment of calcium buffering, the release of pro-apoptotic factors, among others, that inevitably contribute to neuronal loss. A large body of evidence implicates NMDAR-mediated excitotoxicity as a central mechanism in the pathogenesis of many neurodegenerative diseases, including amyotrophic lateral sclerosis (ALS), Alzheimer’s disease (AD), and epilepsy. In this review article, we explore different causes and consequences of excitotoxicity, discuss the involvement of NMDAR-mediated excitotoxicity and its downstream effects on several neurodegenerative disorders, and identify possible strategies to study new aspects of these diseases that may lead to the discovery of new therapeutic approaches. With the understanding that excitotoxicity is a common denominator in neurodegenerative diseases and other disorders, a new perspective on therapy can be considered, where the targets are not specific symptoms, but the underlying cellular phenomena of the disease.

## Excitotoxicity: What Is it, Where Does it Come From, How Does it Live?

### The Good, the Bad, and the Ugly: General Definition and Summary of Consequences

**Figure 1:**
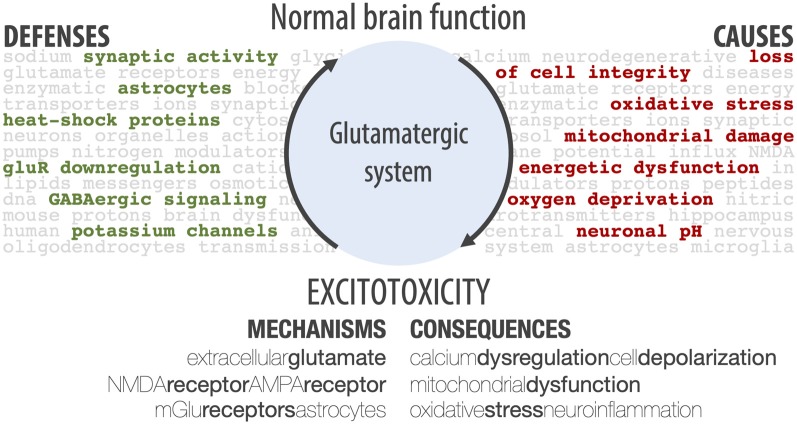
Schematic summary of different aspects of excitotoxicity, including the main cellular defenses against it, the main causes, the players involved in its mechanisms, and general cellular consequences. The shift from normal brain function to an excitotoxic state is brought about by the main causes, and this shift is avoided by the main cellular defenses. If the defenses fail, excitotoxicity is amplified by different mechanisms, causing cellular consequences.

When looking into the glutamatergic system within the central nervous system, one can see three sides of the same biological phenomenon. First, there is The Good: the glutamatergic system is essential for brain functioning. Indeed, with 40% of glutamatergic synapses in the central nervous system (Fairman and Amara, 1999), glutamate is essential for neuronal communication, as well as for higher-level functions, such as learning and memory. Then, there is The Bad: since glutamate plays such an important role in the brain, dysregulation of the glutamatergic system has long been implicated as a key step in the pathophysiology of neuronal death (Bano et al., 2005). Finally, there is The Ugly: excitotoxicity. This phenomenon, an important aspect of glutamatergic dysregulation, describes the toxic actions of excitatory neurotransmitters, mainly glutamate, that ultimately lead to neuronal death (Connolly and Prehn, 2015). While glutamate does not directly kill neurons, the exacerbated or prolonged activation of glutamate receptors starts a cascade of neurotoxicity (Lipton, 2008; Vincent and Mulle, 2009), which includes cationic influx, mitochondrial dysfunction, energetic and oxidative stress, and overproduction of reactive oxygen species (ROS; Connolly and Prehn, 2015; Prentice et al., 2015). Here, we review the main factors involved in excitotoxicity, as summarized in Figure 1, and their role in neurodegenerative diseases and epilepsy.

### If These Walls Could Talk‥. About Excitotoxicity: Historic Review

The term “excitotoxicity” makes its debut in 1969 in a study by Olney where, for the first time, cell death was observed as a consequence of exposure to glutamate or aspartate (Olney, 1969). This type of excitotoxicity can be divided into three steps. It starts with the induction, which begins with the overactivation of ionotropic glutamate receptors (iGluR), such as N-methyl-D-aspartic acid receptors (NMDAR), α-amino-3-hydroxy-5-methylisoxazole-4-propionate receptors (AMPAR) and kainate receptors (KAR). NMDAR display a high permeability to both sodium and calcium (McBain and Mayer, 1994), while AMPAR and KAR activation contributes mainly to sodium influx. This ionic influx is followed by water influx, leading to cell swelling and mitochondrial dysfunction. The final step of induction is the activation of metabotropic glutamate receptors (mGluR), activating pathways regulated by diacylglycerol and inositol 1,4,5-triphosphate (Zivin and Choi, 1991). Amplification and expression are the following stages. These consist of exacerbated metabolic activity, further disruption of ion gradients, elevated electrical neuronal activity, and mitochondria dysfunction (Zivin and Choi, 1991).

Other than this classification system for excitotoxicity, this phenomenon can be further categorized as strong or weak excitotoxicity (Albin and Greenamyre, 1992). Strong excitotoxicity refers to the classical phenomenon described by Olney (1969), where excitotoxicity is elicited by direct exposure to glutamate or another excitotoxic compound. In strong excitotoxicity, the calcium influx is prolonged, there is depolarization of both cell and mitochondria membranes, excessive consumption of NAD(P)H, and an established bioenergetic failure. Furthermore, cell death is generally brought on by necrosis (Castilho et al., 1998; Connolly and Prehn, 2015). On the other hand, weak excitotoxicity results from a prolonged alteration of glutamate receptor function, possibly impacting membrane potential and cellular metabolism and increasing cellular sensitivity to the toxic actions of glutamate (Albin and Greenamyre, 1992). In this scenario, neurons are transiently able to restore calcium homeostasis, membrane potential, and internal ATP and NAD(P)H pools (Ward et al., 2000; Weisová et al., 2009). These neurons can then undergo delayed apoptosis, characterized by delayed calcium dysregulation, nuclear compression, and cellular contraction (Ankarcrona et al., 1995; Bonfoco et al., 1995; Ward et al., 2007; D’Orsi et al., 2012). This is accompanied by a loss of membrane potential, ATP depletion, and overproduction of ROS (Luetjens et al., 2000; Vesce et al., 2004).

### A Glutamatergic Mind: Glutamate in Physiological and Excitotoxic Conditions

In central nervous system synapses, glutamate clearance is achieved by diffusion and transporter uptake, mainly into neighboring glial cells (van den Berg and Garfinkel, 1971; Benjamin and Quastel, 1972; Hertz et al., 1999), and is practically independent of enzymatic breakdown (Watkins and Evans, 1981).

It should however be noted that glutamatergic synapses are complex—their morphological characteristics (number of release sites, the existence of dendritic spines, etc.) can differ greatly and synapses in different neural circuitries exhibit different time courses of synaptic communication (Jonas, 2000). Furthermore, the exact time course of glutamate dynamics can be altered by the microanatomical synaptic properties (synaptic contact size, complexity of synaptic morphology, glial wrapping, etc.) and by the density of glutamate transporters (Jonas, 2000).

Even taking all this into account, during a synaptic event, the peak concentration of glutamate in the synapse can be estimated to be 1.1 mM (1.0–1.5 mM), with a decay time constant of 1.2 ms (0.70–2.0 ms; Clements et al., 1992), while in extrasynaptic locations, the peak concentration can reach 190 μM (Dzubay and Jahr, 1999). On the opposite side, the baseline concentration of synaptic glutamate (with no synaptic event occurring) is thought to vary between 25 nM (Herman and Jahr, 2007) and 600 nM (Mark et al., 2001), depending on the study. This basal concentration is unaltered by synaptic activity, being controlled solely by glutamate transporters (Herman and Jahr, 2007). Furthermore, this basal concentration does not activate glutamate receptors (Trussell and Fischbach, 1989; Patneau and Mayer, 1990; Conn and Pin, 1997) nor interferes with neuronal excitability (Herman and Jahr, 2007).

In excitotoxicity, the time course of glutamate dynamics can be affected in several different manners. The synaptic concentration of glutamate can rise above its usual peak concentration (above 1.1 mM), although this value might plateau due to complete depletion of presynaptic glutamate, or this peak concentration can be maintained for an excessive amount of time, which is translated to an increase in the decay time, or even the baseline concentration of glutamate may be increased, regardless of depolarization-induced release. Regarding this last scenario, it is known that neuronal excitotoxic injury occurs with baseline concentrations of glutamate of 2–5 μM (Mark et al., 2001).

### Glutamate and the Depolarization Factory: Glutamate Receptors in Excitotoxicity

Glutamatergic neurotransmission is performed through iGluR and mGluR. The iGluR are ligand-gated ion channels permeable to various cations, namely sodium, potassium, and calcium, that produce excitatory glutamate-evoked currents, while mGluR are G protein-coupled receptors that control cellular processes *via* G protein signaling cascades (Reiner and Levitz, 2018). The main characteristics of these receptors are summarized in Table 1 and reviewed in detail in this section.

**Table 1:** Summary of glutamate receptors’ characteristics, including their synaptic location, relationship with calcium, and involvement in excitotoxicity.

Receptor type	General description	Receptor subtype	Location	Calcium involvement	Involvement in excitotoxicity
Ionotropic	Ligand-gated ion channels	NMDA	Synaptic and extrasynaptic	Permeable	sNMDA: neuroprotection, cell survival
	Excitatory glutamate-evoked currents				eNMDA: pro-death pathways, excitotoxicity, neuronal dysfunction
		AMPA	Post-synaptic	Permeable if lacking GluA2 subunit	Activation alleviates NMDAR Mg^2+^ block, cell depolarization
		KA	Pre- and post-synaptic	Mostly impermeable	
		
Metabotropic	GPCR	mGluR1/5	Peri- and extrasynaptic	Ca^2+^ release from internal stores	Activation of the IP_3_/Ca^2+^ signaling pathway
	Cellular processes *via* G protein signaling cascades				Delayed cell death

iGluR can be divided into three functional classes such as NMDAR, AMPAR, and KAR (Hansen et al., 2017). While all iGluR is in the first line of the excitotoxic response, NMDAR has been pinpointed as the main culprit in glutamate-induced neurotoxicity, due to their permeability to calcium ions. NMDAR exhibits a voltage-dependent magnesium-blockade, high permeability to calcium, and requires simultaneous binding of glutamate and a co-agonist, such as glycine and D-serine, for activation (Guo et al., 2017). NMDAR are tetrameric structures composed of GluN1, GluN2A-D and GluN3A-B subunits that form a central ion channel pore. Diversity in NMDAR subunits and assembly results in different receptor subtypes with distinct functional properties, including different channel kinetics, channel opening probability, and conductance. Therefore, these differences in subunit composition can impact both synaptic plasticity and neuronal function. Previous literature reported that GluN2A-containing receptors present faster kinetics, while GluN2B-containing receptors present slower opening, closing, and glutamate unbinding, indicating that NMDAR containing GluN2A open more reliably and with faster kinetics than NMDAR containing GluN2B subunits (Erreger et al., 2005). These data suggest that NMDAR containing GluN2A is more likely to sense rapid glutamate transients in the synapse and open with a high probability, while NMDAR containing GluN2B seems to be set to sense basal levels of glutamate and open with much lower probability. Moreover, it is known that NMDAR subunit composition also determines single-channel conductance and blockage by magnesium ions (Kuner and Schoepfer, 1996; Brimecombe et al., 1997; Cull-Candy et al., 2001), where both GluN2A- or GluN2B-containing receptors display a high ionic conductance and stronger magnesium block. In mature neurons, GluN2A subunits are predominantly synaptic (sNMDAR), whereas GluN2B-containing receptors are mostly extrasynaptic (eNMDAR; Rönicke et al., 2011). In rat hippocampal slices, the amount of eNMDAR has been estimated to be 36% of that of sNMDAR (Harris and Pettit, 2007) and it is known that D-serine mainly binds to sNMDAR, while glycine preferentially binds to eNMDAR (Papouin et al., 2012). Furthermore, previous studies reported a neuroprotective role for sNMDAR and a neurotoxic role for eNMDAR by receptor-mediated influx of calcium (Hardingham et al., 2002; Hardingham and Bading, 2010; Samson et al., 2016). Moreover, it has been proposed that the activation of NMDAR containing GluN2A subunits stimulates signaling cascades associated with neuroprotection and regulates survival, while stimulation of NMDAR containing GluN2B subunits leads to the activation of excitotoxic pathways, leading to neuronal death (Zhou et al., 2013). sNMDAR (mainly enriched with GluN2A subunits) activation promotes neuroprotection *via* changes in gene expression that have multiple effects within the cell. Stimulation of sNMDAR is known to result in the enhancement of antioxidant defenses, promoting the transcription of pro-survival factors, including cAMP-regulatory element-binding protein (CREB; Hardingham and Bading, 2010), that results in the transcription of brain-derived neurotrophic factor (BDNF), essential for neuronal survival. sNMDAR activity is also implicated in the generation of anti-apoptotic effects, including the inactivation of pro-death transcription factors, such as forkhead box protein O and p53 (Dick and Bading, 2010). In contrast, stimulation of eNMDAR (mainly enriched with GluN2B subunits) preferentially induces pro-death effects, such as the shut-off of CREB pathway, blocking BDNF expression (Hardingham et al., 2002); the inactivation of extracellular signal-regulated kinases 1 and 2, which are necessary for BDNF function on spines (Hardingham et al., 2002); activation of forkhead box protein O and calpains, with consequent cleavage of the striatal enriched tyrosine phosphatase, which prevents this phosphatase from inhibiting p38 mitogen-activated protein kinase; and oxidative stress with subsequent neurodegeneration (Hardingham et al., 2002; Hardingham and Bading, 2010). Hence, while sNMDAR has a neuroprotective role, eNMDAR preferentially initiates cell death pathways (Hardingham and Bading, 2010). It is known that low levels of eNMDAR activity have no effects on neuronal survival, while high levels of eNMDAR stimulation enhance cell death pathways and exacerbate neurodegenerative processes, consequently reducing neuronal survival (Hardingham and Bading, 2010). eNMDAR can also be activated by mechanical stimuli and aspartate. Indeed, the GluN2B subunit has been reported to play a role in the mechanosensitive activation of NMDAR (Maneshi et al., 2017). On the other hand, although with lower affinity than glutamate, aspartate can bind and activate NMDAR (Chen et al., 2005). Aspartate levels are severely increased following traumatic brain injury (Palmer et al., 1994; Amorini et al., 2017) and, when present in excessive levels extrasynaptically, are associated with excitotoxicity (Choi et al., 1989). In summary, while sNMDAR signaling is associated with the suppression of pro-apoptotic transcription factors (Dick and Bading, 2010), eNMDAR stimulation increases the activity of pro-apoptotic factors, leading to oxidative stress and cell death (Hardingham et al., 2002; Hardingham and Bading, 2010; Parsons and Raymond, 2014). The activation of these pro-apoptotic factors can affect gene activity regulation of BDNF and vascular endothelial growth factor (Hardingham et al., 2002), essential for the maintenance of synaptic connectivity and architecture (Bading, 2013). Hence, the excessive intracellular calcium levels resulting from the excessive eNMDAR stimulation lead to excitotoxic cell death and contributes to neuronal injury.

Intriguingly, there are conflicting pieces of evidence regarding NMDAR involvement in excitotoxicity. Some studies state that NMDAR has a dichotomic effect in excitotoxicity, where activation of sNMDAR counteracts excitotoxicity and activation of eNMDAR is the main contributor to the excitotoxic cascade (Jia et al., 2015). Other authors propose that NMDAR-induced excitotoxicity requires overactivation of both sNMDAR and eNMDAR (Zhou et al., 2013, 2015). Finally, other studies even suggest neurotoxicity could be solely dependent on sNMDAR activity, since silencing sNMDAR can act as a neuroprotective approach against NMDA-induced excitotoxicity, while inhibiting eNMDAR does not have any protective effect, questioning the role of these receptors in neurotoxicity (Papouin et al., 2012).

AMPAR are tetrameric ionotropic glutamate receptors formed by GluA1–4 subunits. These receptors can be either hetero- or homotetramers and, depending on subunit composition, display different calcium permeability (Pál, 2018), with the GluA2 subunit controlling AMPAR calcium permeability. Assemblies of highly calcium-permeable AMPAR have been implicated in excitotoxicity. Moreover, the GluA2 subunit can also be subjected to RNA editing, with the conversion of a glutamine codon into an arginine one, and AMPAR is calcium-permeable if they contain the unedited GluA2 subunit or if they lack the GluA2 subunit (Wright and Vissel, 2012). Given their high calcium permeability, AMPAR lacking GluA2 is thought to contribute to excitotoxic cell death (Wright and Vissel, 2012).

KAR is composed of GluR5-7 and KA1-2 subunits. KAR properties are similar to AMPAR in that they allow ion flux directly following glutamate exposure, and are mostly impermeable to calcium. Although AMPAR is localized mostly in the postsynaptic membrane, several studies have shown that KAR may be localized in both pre- and post-synaptically (Chittajallu et al., 1996; Castillo et al., 1997). Post-synaptically, KAR, and AMPAR have a similar function in alleviating the magnesium block in NMDAR. This phenomenon leads to an exacerbation of NMDAR activation under glutamate excess.

mGluR are probably the most diverse receptor family of the central nervous system (CNS). The mGluR1 and mGluR5 subtypes are located in the peri- and extrasynaptic neuronal regions (Ferraguti et al., 2008). They are coupled to G_q_ protein and exert their actions *via* the inositol trisphosphate/calcium signal pathway, being consequently able to stimulate calcium release from neuronal stores, thus triggering delayed cell death (Pál, 2018).

### The Gatekeepers of Homeostasis: Astrocytes

In the forebrain, it has been shown that astrocytes are responsible for about 90% of the glutamate clearance from the synaptic cleft. During synaptic transmission, only approximately 20% of synaptically-released glutamate reaches the postsynaptic glutamate receptors, while the remainder can reach the extrasynaptic space (Kojima et al., 1999). Due to the significant role of astrocytes in glutamate re-uptake, impairment of astrocytic glutamate transporters leaves neurons highly susceptible to excitotoxicity (Rothstein et al., 1996). During an acute insult, astrocytes can prevent excitotoxicity by removing extracellular glutamate with high-affinity sodium-dependent glutamate transporters also known as excitatory amino acid transporters (EAAT). These proteins can clear glutamate from the extracellular space into cells, where it can be metabolized or recycled. Several subtypes of EAAT have already been pharmacologically identified. These transporters were divided into four subtypes in rats (GLAST, GLT-1, EAAC1, and EAAT4) and five in humans (EAAT1-5), and can be found in neurons (EAAT3 or EAAC1 and EAAT4) and astrocytes (EAAT1 or GLAST and EAAT2 or GLT-1; Danbolt, 2001; Crino et al., 2002; Beart and O’Shea, 2007; Sarac et al., 2009; Gonçalves-Ribeiro et al., 2019). Additionally, although glutamate transporters activity may come mostly from (if not exclusively) from astrocytes, the role of glutamate transporters in neurons still needs to be further explored. Indeed, neuronal glutamate transporters knockout does not influence in terms of preventing excitotoxicity, since mice with this knockout show no differences in survival, weight gain, and seizure activity compared to wild-type. On the other hand, synaptosomes prepared from these knockout mice showed a reduction of glutamate activity by 40% (Petr et al., 2015), suggesting that the role of neuronal glutamate transporters is yet to be revealed.

Furthermore, astrocytes are responsible for the maintenance of glutamate homeostasis by sustaining its synthesis, uptake and release *via* the glutamate-glutamine cycle (van den Berg and Garfinkel, 1971; Benjamin and Quastel, 1972; Ottersen et al., 1992). Through this cycle, synaptically released glutamate is predominantly taken up into astrocytes, where it is amidated to glutamine by the astrocyte-specific enzyme glutamine synthetase (Norenberg and Martinez-Hernandez, 1979). Glutamine is then released to the synapse and uptake by adjacent neurons, where it is converted to glutamate and γ-aminobutyric acid (GABA), which are then repackaged into vesicles and again released in the synapse as neurotransmitters (Allen, 2014; Rodríguez-Arellano et al., 2016).

In a different perspective, astrocytes are hypothesized to be the main effectors of glycolysis to produce lactate that is then transferred to neurons through the astrocyte-neuron lactate shuttle. This metabolic link between neurons and astrocytes is because neurons are highly energy-demanding cells but are unable to perform the citric acid cycle without an external supply of lactate, due to the lack of the essential enzyme pyruvate carboxylase (Schousboe et al., 1997; Magistretti and Allaman, 2015). Since astrocytic glutamate re-uptake is electrogenic, for each glutamate molecule entrance, three sodium ions enter the cell, activating the sodium/potassium ATPase. This, in turn, activates the glycolysis pathway in the astrocytes, stimulating the production of the lactate by either increasing glucose uptake or intracellular glycogen breakdown. Produced lactate is later released into the intracellular space by the monocarboxylate transporter 1 expressed in the astrocytes and received by neurons through the monocarboxylate transporter 2 (Stobart and Anderson, 2013). It is later used by the neuron as the carbon source in the Krebs cycle for oxidative phosphorylation. Besides lactate, astrocytes also release citrate, involved in the regulation of neuronal excitability by chelating zinc ions, thus inhibiting NMDAR (Westergaard et al., 1995; Schousboe et al., 1997). Furthermore, although recent literature suggests that neurons are primarily responsible for the synthesis and release of D-serine (Wolosker et al., 2016), several independent studies strongly support that astrocytes are the main source of D-serine, essential for NMDAR function (Henneberger et al., 2010; Bergersen et al., 2012; Martineau et al., 2013; Sultan et al., 2015).

## The Butterfly Effect: Detailed Look Into Excitotoxicity-Induced Dysfunctions

### One Ion to Rule Them all: The Central Role of Calcium to Propagate Glutamate Toxicity

**Figure 2:**
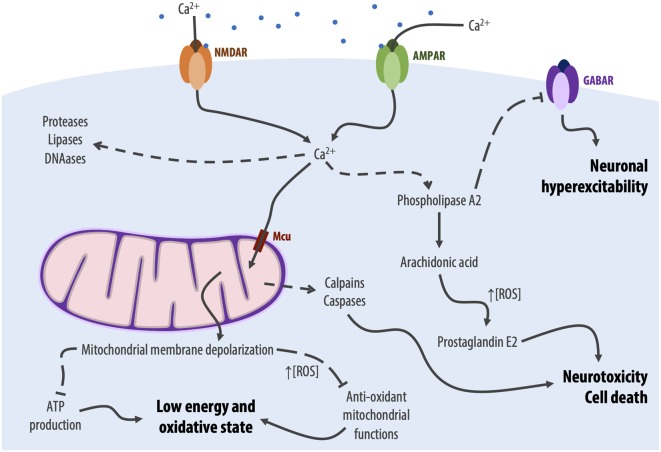
Graphical depiction of the main calcium-dependent processes involved in excitotoxicity, leading to oxidative stress and cell death. Dotted lines with end arrows represent activation, dotted lines with T ends represent inhibition. Mcu, mitochondrial calcium uniporter.

One of the first consequences of excessive activation of glutamate receptors, namely NMDAR, is a sustained influx of calcium into the neuron (Mehta et al., 2013). Calcium has long been identified as a key player in glutamate neurotoxicity, as summarized in Figure 2. Specifically, early studies proved that scavenging extracellular calcium would decrease excitotoxicity-induced neuronal degeneration, while removal of other cations would not (Berdichevsky et al., 1983; Choi et al., 1988). This influx synergizes with the release of calcium from intracellular stores, such as the mitochondria and the endoplasmic reticulum, due to membrane damage caused by the disruption of ionic gradients (Mehta et al., 2013).

Together, these phenomena lead to a striking increase in the intracellular calcium concentration, which can activate enzymes that degrade proteins, lipids, and nucleic acids (Berliocchi et al., 2005), as well as enzymes involved in arachidonic acid pathways, such as phospholipase A2, cyclooxygenase-2, and lipoxygenases. These latter enzymes, when activated, lead to the production of arachidonic acid and its conversion into prostaglandins, leukotrienes, and thromboxanes with the concomitant production of ROS (Freeman and Crapo, 1982; Pazdernik et al., 1992; Murphy et al., 1994). Among these molecules, prostaglandin E2 has been shown to have a dual role in excitotoxicity: at low concentration (nanomolar range), prostaglandin E2 presents a neuroprotective effect (Akaike et al., 1994); at higher concentrations (micro- to millimolar), as seen in the calcium-induced arachidonic acid cascade, prostaglandin E2 contributes to neurotoxicity and cell death (Hewett et al., 2000). Additionally, calcium-induced activation of phospholipase A2 inhibits GABA receptors (GABAR), thus preventing neuronal hyperpolarization and further contributing to excitotoxicity (Hamano et al., 1996).

Finally, the calcium dysregulation also activates ATP-dependent ion pumps, to counteract the cationic influx, such as the sodium/potassium and calcium ATPases, which will drain all available ATP, creating a low-energy neuronal state (Connolly et al., 2014; Surin et al., 2014).

### Eternal Dysfunction for the Mitochondrial Minds: Mitochondrial Damage

Another large contributor to glutamate neurotoxicity are mitochondria (Figure 2), which are essential in bioenergetic homeostasis and calcium signaling regulation (Mehta et al., 2013). Indeed, following the excitotoxicity-induced calcium influx, mitochondria capture cytosolic calcium *via* the mitochondrial calcium uniporter, attempting to maintain a low cytosolical calcium concentration (Qiu et al., 2013). While this process temporarily buffers the intracellular calcium concentration, the excessive cationic uptake eventually leads to the depolarization of the mitochondrial membrane, impairing ATP production and anti-oxidant mitochondrial functions (Nicholls and Budd, 2000; Kushnareva et al., 2005) and leaving neurons in a low-energy and oxidative state with high production of ROS (Mehta et al., 2013). Following this cascade, mitochondrial dysfunction contributes to apoptotic cell death, activating both caspases (Lipton, 2008) and calpains (Caldeira et al., 2014).

### The Wizard of ROS: Oxidative Stress

The excessive activation of glutamate receptors also leads to an imbalance between ROS and their opposing antioxidant forces (Bondy and LeBel, 1993), a phenomenon named oxidative stress. In an excitotoxic scenario, oxidative stress is brought about by a higher intracellular concentration of ROS, which is directly associated with glutamate neurotoxicity (Nicholls, 2004). ROS are then mediators of enzyme inactivation, lipidic peroxidation and consequent membrane damage, DNA alterations, and apoptosis (Floyd, 1999; Lucca et al., 2009).

The excitotoxic production of ROS is related to the activation of the pro-oxidant enzymes xanthine oxidase and NADPH oxidase (Prentice et al., 2015) and mitochondrial dysfunction. Interestingly, NADPH oxidase, and not mitochondria, has been identified as the major source of ROS following glutamate exposure (Brennan et al., 2009).

Additionally, studies have demonstrated that glutamate binding to NMDAR results in the production of nitric oxide (NO) as a consequence of nitric oxide synthase (NOS) activation by calcium influx, which is spatially linked to NMDAR *via* the postsynaptic density protein of 95 kDa (Aarts et al., 2002; Zhou et al., 2010; Jones, 2011). It has also been observed that an increase in NO concentration can trigger biochemical pathways that contribute to neuronal death and cognitive impairment (Díaz et al., 2010), since NO can react with the superoxide anion forming peroxynitrite, known to lead to the formation of 3-nitrotyrosine (Butterfield and Kanski, 2001). NO is then responsible for protein degradation by nitration and oxidation, lipid peroxidation, and DNA damage (Jia et al., 2015). Protein nitration results in the dysfunction of several proteins such as superoxide dismutase (SOD), actin, and tyrosine hydroxylase, and can interfere with cell signaling pathways mediated by tyrosine phosphorylation (Butterfield and Stadtman, 1997), contributing to the intracellular signaling dysregulation.

## We Need to Talk About Excitotoxicity: Excitotoxicity as a Consequence of Other Phenomena

A possible trigger for excitotoxicity is the physical damage of a neuron. In physiological conditions, the extracellular glutamate concentration is around 0.01% of the intracellular concentration, not reaching the threshold for post-synaptic neuronal activation which maintains the glutamate pools in an inactive state (Mehta et al., 2013). However, this percentage can quickly increase if a damaged neuron releases all its glutamate content into the extracellular space, leading to excitotoxicity in neighboring neurons (Mehta et al., 2013).

Oxidative stress is one of the main consequences of glutamate-induced neurotoxicity. However, it is not possible to define a unidirectional cause/effect relationship between the two phenomena, since oxidative stress and excessive intracellular ROS can also induce excitotoxicity by stimulating extracellular glutamate release (Pellegrini-Giampietro et al., 1990) and releasing calcium from mitochondria into the cytosol (Richter and Kass, 1991). In another perspective, it has already been shown that astrocytic glutamine synthetase is especially susceptible to ROS-induced inactivation, which compromises the whole glutamate-glutamine cycle and contributes to an increase in extracellular glutamate concentration and consequent excitotoxicity (Schor, 1988). Additionally, the presence of ROS has been shown to decrease glutamate transporter activity, impairing synaptic clearance of glutamate further contributing to the increase in extracellular glutamate concentration (Anderson and Swanson, 2000).

Oxygen deprivation can also induce excitotoxicity through different mechanisms. In a straight forward manner, hypoxic-ischemic states directly stimulate glutamate release, increasing the extracellular glutamate concentration to neurotoxic levels (Prentice et al., 2015). Additionally, oxygen deprivation leads to energetic stress by impairing mitochondrial oxidative phosphorylation and, consequently, blocking ATP production (Doyle et al., 2008). This blockage leads to the depletion of intracellular ATP, which prevents the reuptake of glutamate, creating an excitotoxic concentration of extracellular glutamate (Rossi et al., 2000).

In a different perspective, while mitochondrial damage is one of the main consequences of excitotoxicity, mitochondria can also be originators of excitotoxicity. Mitochondrial toxins, for instance, can cause energetic impairment, preventing ATP production (Dong et al., 2009). Over time, cellular ATP pools are then depleted, with a concomitant decline in the activity of the sodium/potassium ATPase, which will depolarize the neuronal membrane (Mehta et al., 2013). By itself, this depolarization can render neurons more prone to firing action potentials (Dutta and Trapp, 2011). It, however, also affects NMDAR by removing their voltage-dependent magnesium-block, lowering their activation threshold so that non-excitotoxic glutamate concentrations become capable of inducing an excitotoxic response (Stavrovskaya and Kristal, 2005).

Finally, the physiological brain pH is estimated to be pH 7.2–7.3 in rats (Siemkowicz and Hansen, 1981), and pH 7.33 in humans (Mutch and Hansen, 1984). However, some diseases or disorders can drastically alter the CNS pH, inducing either acidosis or alkalosis. For instance, anaerobic conditions of ischemia promote the alteration of the metabolism of the cells from aerobic oxidation of glucose to anaerobic glycolysis. This alteration in metabolism produces lactate and protons, which is responsible for acidosis (Li et al., 2016). Interestingly, a curious phenomenon occurs in acidosis. While severe acidosis (pH < 6.4) contributes to excitotoxicity through a loss of ionic gradients (Kraig et al., 1987) and activation of acid-sensing ion channels, which intensifies excitotoxicity by providing another entry for calcium (Waldmann et al., 1997; Cheng et al., 2018; Qiang et al., 2018), mild acidosis (pH 6.5–7.0) appears to partly prevent excitotoxicity (Simon et al., 1993), as well as glutamate-induced neuronal death (Tombaugh and Sapolsky, 1990). This phenomenon occurs due to the inhibitory effects of protons on NMDAR activation (Tang et al., 1990). On the other end of the spectrum, alkalosis (pH > 8.0) seems to induce neurotoxicity. When compared to acidosis, alkalosis produces a more severe dysfunction that is more difficult to counteract. Indeed, cortical GABAergic neurons appear to be more susceptible to alkalosis than to acidosis (Zhang et al., 2013). And, considering the inhibitory effect of protons on NMDAR, it is possible to conclude that alkalosis leads to an increase in NMDAR activation. Thus, alkalosis contributes to excitotoxicity by both directly stimulating NMDAR, but also by disrupting inhibitory neurotransmission.

## Saving Private Neuron: Cellular Defenses Against Excitotoxicity

During excitotoxicity, neurons mobilize a variety of defenses to decrease the damaging effects of this process, among which are potassium channels, GABA signaling, acid-sensing ion channels, adenosine, and NO (Sapolsky, 2001).

Potassium channels are responsible for limiting and rectifying neuronal excitability during action potentials. The small-conductance calcium-dependent potassium channels (Sah, 1996) are highly calcium-dependent and are quite sensitive to transient increases of cytosolic calcium (Blatz and Magleby, 1987). The small-conductance channels mediate the shift of the excitotoxic calcium mobilization into a protective, hyperpolarizing signal (Madison and Nicoll, 1984; Lancaster and Adams, 1986; Sah, 1996; Honrath et al., 2017). Other key potassium channels are the ATP-dependent potassium channels, whose conductance is enhanced by ATP depletion (Politi and Rogawski, 1991; Riepe et al., 1992), particularly following excitotoxic insults (Trapp and Ballanyi, 1995). At presynaptic sites, ATP-dependent potassium channel activation also inhibits glutamate release (Bancila et al., 2004).

NMDAR-mediated calcium influx also increases sodium/potassium ATPase activity, leading to decreased excitability by stabilizing the resting membrane potential (Marcaida et al., 1996).

Another defense mechanism against excitotoxicity involves the inhibitory neurotransmitter GABA and GABAergic signaling (Bradford, 1995). Also, taurine released from glial cells during insults (Magnusson et al., 1991; Torp et al., 1991), similarly to GABA, also decreases presynaptic neuronal excitability by increasing chloride influx (Huxtable, 1992) through GABA_A_R (O’Byrne and Tipton, 2000; Winkler et al., 2019). Moreover, neuronal networks were shown to offer a fast-acting GluN2A-dependent neuroprotective signaling mechanism, which uses the innate capacity of surrounding neuronal networks to quench excitation, through the recruitment of GABA_B_R (Samson et al., 2016).

Glutamate receptor number can be also decreased by calcium-mediated activation of calpains. Activation of these proteases can result in the proteolysis of both NMDAR and AMPAR (Bi et al., 1996, 1998a,b). Moreover, calcium-dependent activation of calcineurin and calmodulin can inhibit voltage-gated and NMDAR-gated calcium currents, respectively (Vyklický, 1993; Lieberman and Mody, 1994; Ehlers et al., 1996).

During necrotic insults, energy depletion gives rise to adenosine. Adenosine inhibits presynaptic glutamate release, an action that is accomplished through A_1_ adenosine receptors linked by G proteins to both calcium and potassium channels and decreases postsynaptic calcium currents in response to glutamate (Phillis and Wu, 1981; Cunha, 2005). On the other hand, a downstream consequence of glutamatergic excitotoxicity is the generation of NO, which acts intracellularly to inhibit NMDAR activity by nitrosylation (Lipton et al., 1993).

Another response of neurons to cell stress is the expression of heat-shock proteins, which protect against misfolding, conferring resistance to necrotic injury (Yenari et al., 1999). Neurons can also up-regulate and increase the activity of antioxidant agents following necrotic insults, which includes Mn- and Cu/Zn-SOD (Ohtsuki et al., 1993; Fukuhara et al., 1994; Matsuyama et al., 1994), glutathione peroxidase, and catalase (Goss et al., 1997).

Also, recent evidence has shown that synaptic activity can protect primary rat hippocampal neurons against mitochondrial oxidative stress and mitochondrial dysfunction derived from acute excitotoxicity, since synaptic activity can induce the transcriptional repression of the mitochondrial calcium uniporter, leading to a reduction in excitotoxicity associated with mitochondrial calcium overload (Depp et al., 2018).

## A Brief History of ALS: Amyotrophic Lateral Sclerosis

Amyotrophic Lateral Sclerosis (ALS) is a fatal and progressive neurodegenerative disease, characterized by the degeneration of both upper (motor cortex) and lower (spinal cord) motor neurons that result in motor dysfunction and, ultimately, death. While primary symptoms of ALS are associated with motor dysfunction, other areas of the brain may undergo degeneration, with 40–60% of patients showing evidence of different levels of cognitive impairment (Witgert et al., 2010; Ferrari et al., 2011). Although 90% of ALS cases are sporadic with no known cause, in the 10% of familial cases, more than 30 genes have already been implicated (Chen et al., 2013), with mutations in Cu/Zn-SOD1, transactive response DNA-binding protein, fused in sarcoma, chromosome 9 open reading frame 72, and several others (Ajroud-Driss and Siddique, 2015).

**Figure 3:**
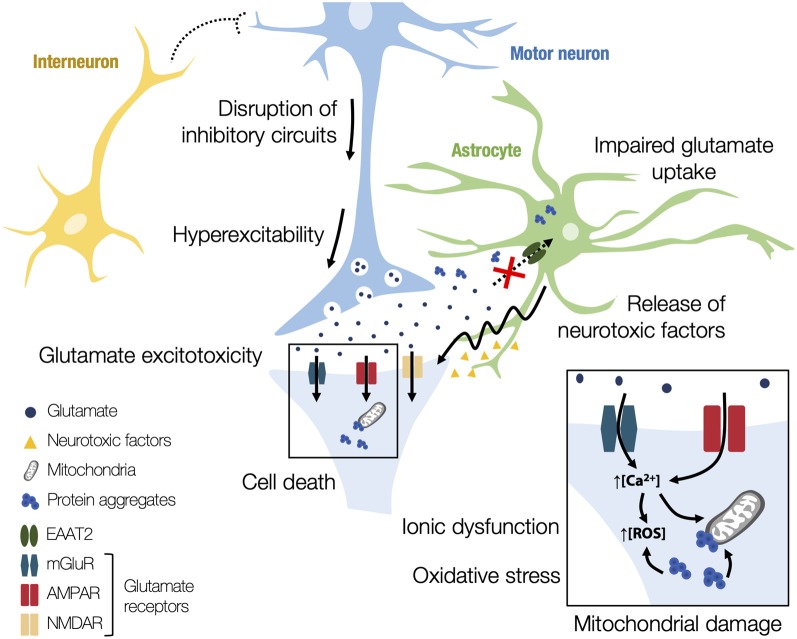
Schematic representation of the alterations in excitatory neurotransmission in amyotrophic lateral sclerosis (ALS). Different pathophysiological mechanisms have been proposed to explain excitotoxicity in ALS. Interneuron alterations are observed in early disease stages, with a loss of cortical and spinal interneurons, leading to the disruption of inhibitory circuits. Consequently, there is an excitation–inhibition imbalance, increasing subsequent excitability and glutamate release to the synaptic cleft. Glutamate-mediated excitotoxicity may happen through an astrocyte-mediated downregulation of excitatory amino acid transporter 2 (EAAT2), which decreases the glutamate uptake from the synaptic cleft and potentiates the excitotoxic effects. Astrocytes in ALS also release neurotoxic factors that trigger changes to motor neuron glutamate receptors and render them susceptible to excitotoxicity, furthering neurodegeneration. Moreover, the excessive firing and the dysregulated calcium influx through atypical glutamate receptors results in an ionic dysfunction in motor neurons. The excessive entry of calcium into motor neurons results in mitochondrial overload and in the generation of reactive oxygen species (ROS), which ultimately causes oxidative stress. The presence of protein aggregates in mitochondria can also lead to alterations in normal cell metabolism, increasing the susceptibility to glutamatergic overstimulation as well as the activation of apoptotic pathways.

Despite numerous studies demonstrating the involvement of several altered signaling pathways, the pathogenetic mechanisms behind ALS are still unclear. Indeed, it seems that, in ALS, neurodegeneration is the product of a combination of different concomitant mechanisms. Understanding the cause of motor neuron degeneration is critical for unraveling ALS pathogenesis and there are mainly two hypotheses to explain the origin of the disease (Kiernan et al., 2011). The first one is the dying-forward hypothesis, which proposes an anterograde degeneration of motor neurons *via* glutamate excitotoxicity from the cortex. The second one is the dying-back hypothesis, which suggests that ALS may start distally at the nerve terminal or the neuromuscular junction, progressing towards the cell body. Despite the numerous hypotheses proposed, alterations in excitatory neurotransmission appear to have a key role in disease progression, mediated by increased susceptibility to excitotoxicity, as schematically depicted in Figure 3 (Bae et al., 2013). Physiological studies have demonstrated cortical hyperexcitability in patients with both sporadic and familial ALS before the onset of symptoms (Vucic and Kiernan, 2013). In addition to cortical hyperexcitability, peripheral axons also present changes in their excitability in ALS patients (Vucic and Kiernan, 2006). Changes in neuronal activity can also lead to morphological alterations. In the SOD1^G93A^ mouse model, upper motor neurons display reductions in dendritic length and spine density, suggesting a homeostatic response to heightened pre-synaptic activity or even a stressed state of these neurons (Fogarty et al., 2016; Saba et al., 2016). It is however still unclear whether hyperexcitability is one of the causes of motor neuron degeneration or a compensatory mechanism resulting from motor neuron degeneration.

One of the first proposed mechanisms underpinning neurodegeneration in ALS was glutamate-mediated excitotoxicity (Bendotti and Carrì, 2004). One suggested way in which glutamate excitotoxicity may occur is through a decrease in the levels of the transporters responsible for the removal of glutamate from the synaptic cleft. In ALS, a likely trigger for neuroinflammation is the motor neuronal and astrocytic release of misfolded proteins, like aberrant SOD1, and other toxic molecules. This release stimulates the activation of microglia, unleashing pro-inflammatory and neurotoxic actions (Appel et al., 2011; Zhao et al., 2013; Brites and Vaz, 2014; Pinto et al., 2017), which dysregulate the communication between motor neurons and glial cells, compromising neuronal homeostasis. Microglia are considered the immune cells of the brain and may adopt different polarized activated phenotypes, with the M1 and M2 being the most accepted. The classical M1 phenotype is associated with the release of pro-inflammatory molecules and activation of receptors, and the M2 phenotype is related to the secretion of anti-inflammatory mediators and growth factors. However, recent studies point to the coexistence of different heterogeneous states and mixed phenotypes (Tang et al., 1990; Pinto et al., 2017). In ALS, microglia function changes along with disease progression, displaying the M2 anti-inflammatory phenotype at early stages and switching to the M1 activated subtype as the disease progresses (Zhao et al., 2013; Gravel et al., 2016). Activated microglia contribute and enhance motor neuron death, and can even acquire a distinct and impaired phenotype at the end-stage that accelerates disease progression (Nikodemova et al., 2014; Pinto et al., 2017). In addition to neuroinflammation, glial cells also play a role in glutamate-mediated excitotoxicity. Specifically, astrocyte-mediated downregulation of EAAT2, leading to a decrease in glutamate uptake and subsequent potentiation of excitotoxic effects, has been reported in both ALS patients and SOD1^G93A^ mice (Howland et al., 2002), as well as in the TDP-43 rat model (Tong et al., 2013), correlating with regions of motor neuron loss (Sasaki et al., 2000). Thus, a decrease in the level of these transporters may lead to the accumulation of glutamate in the extracellular space, resulting in postsynaptic glutamate receptor overstimulation and consequent excitotoxic effects (Lin et al., 1998; Zarei et al., 2015). Furthermore, not only have astrocytes in ALS been reported to release lower levels of neurotrophic factors, but they have been shown to release neurotoxic factors that play a role in furthering neurodegeneration (Komine and Yamanaka, 2015; Cunha et al., 2018; Gomes et al., 2019). Moreover, it has been shown that, in ALS patients carrying *SOD1* gene mutations, there is a decrease in motor neuron levels of calcium-binding proteins, which—by decreasing calcium buffering in the cytoplasm—may exacerbate excitotoxicity (Bernard-Marissal et al., 2012; Mattson, 2013). In the SOD1^G93A^ mouse model, overexpression of EAAT2 delays disease onset but not death (Guo et al., 2003) and fails to prevent loss of phrenic nerve motor neurons or rescue respiratory function (Li et al., 2015). Several pathways have been implicated in the modulation of EAAT2 levels, such as tumor necrosis factor-α (TNF-α) and downstream nuclear factor κ-B signaling (Boycott et al., 2008). Recently, membralin, an endoplasmic reticulum membrane protein, was also shown to have a role in EAAT2-mediated glutamate excitotoxicity in ALS. Membralin levels are reduced in human ALS spinal cord and SOD1^G93A^ mouse models and its deletion suppresses EAAT2 expression through a TNF-α/TNF receptor 1/nuclear factor κ-B pathway. Overexpression of membralin in astrocytes was shown to increase EAAT2 expression and improve motor neuron survival (Jiang et al., 2019).

However, defects in the clearance of glutamate do not seem to be the only origin of excessive extracellular glutamate. In the SOD1^G93A^ mouse model, activation of Group I mGluR or the GABA/glycine heterotransporter abnormally increases the release of glutamate (Raiteri et al., 2003; Giribaldi et al., 2013; Milanese et al., 2015). Recently, Bonifacino and colleagues (Bonifacino et al., 2019) demonstrated that mGluR are overexpressed in the spinal cord of SOD1^G93A^ mice at pre-symptomatic stages and that their function is altered early on in the disease, suggesting that it can represent a cause rather than a consequence of disease progression. Group I mGluR may be potential targets for preventing excitotoxicity in ALS since it has been shown that treatment with an antagonist attenuated cell death, delayed the onset of motor symptoms, and slightly prolonged survival in SOD1^G93A^ mice (Rossi et al., 2008).

Moreover, calcium permeability of AMPAR is largely determined by the presence of the GluA2 subunit and it has been shown that mutant SOD1 astrocytes secrete factors that lower the expression of this subunit in motor neurons, consequently leading to AMPAR-mediated excitotoxicity and cell death (Van Damme et al., 2005, 2007). Moreover, fused in sarcoma-ALS astrocytes trigger changes to motor neuron AMPAR that render them susceptible to excitotoxicity (Kia et al., 2018) and patients with the chromosome 9 open reading frame 72 mutations appear to have increased vulnerability to AMPAR-mediated excitotoxicity (Selvaraj et al., 2018).

Besides glutamate-mediated excitotoxicity, excitotoxicity may arise from an altered regulation by interneurons. Interneurons are one of the main regulators of neuronal signaling and the majority in the cortex is inhibitory, using GABA or glycine as a neurotransmitter. In healthy individuals, a subthreshold stimulus of the motor cortex generally leads to the activation of inhibitory GABAergic interneurons, thus reducing subsequent excitability, in a process called short intracortical inhibition (Wagle-Shukla et al., 2009). In ALS patients, however, cortical inhibition is impaired, with a reduced or complete absence of short intracortical inhibition. Importantly, the reduction of short intracortical inhibition was shown to be an adverse prognostic factor in ALS (Shibuya et al., 2016). Several post-mortem reports indicate a loss of cortical and spinal interneurons, in addition to motor neuron losses (Stephens et al., 2006). These observations are seen in different mouse models that also exhibit interneuron alterations in early disease stages. The wobbler mouse model displays hippocampal hyperexcitability, together with a reduced number of interneurons (Thielsen et al., 2013). Also, in the SOD1^G93A^ mouse model, decreases in calretinin interneurons and subsequent increases in parvalbumin interneurons in motor and somatosensory cortex may be compensatory changes to improve excitation–inhibition balance (Chung et al., 2005; Minciacchi et al., 2009). In the spinal cord of the SOD1^G93A^ mice, interneurons degenerate before the loss of motor neurons (Martin et al., 2007). Further studies on a mutant SOD1 zebrafish model revealed that interneurons are the first to exhibit neuronal stress and, once more, that the reduction of inhibitory currents or interneurons preceded any defects in motor neurons (McGown et al., 2013).

Another known mechanism involved in ALS neurodegeneration is oxidative stress. The major enzyme involved in the prevention of oxidative damage is the Cu/Zn-SOD1 enzyme. Congruently, mutations in the *SOD1* gene—resulting in either a decrease/loss or a dominant gain of function—have been found to contribute to cytotoxicity. In line with this, a study demonstrated that the cerebrospinal fluid, serum, and urine samples of ALS patients had increased levels of free radicals and concomitant oxidative stress (Zarei et al., 2015). Another source of oxidative damage in mitochondrial dysfunction. The existence of alterations is normal cell metabolism due to misfolded mutant SOD1 deposits in mitochondria has been reported in both ALS patients and mouse models. Furthermore, dysfunctions in energy, alterations in the triggering of apoptotic signals, disruptions in mitochondrial transport along axons, and atypical production of ATP and ROS have been also associated with ALS (Pasinelli et al., 2000; Mattiazzi et al., 2002; Menzies et al., 2002; Damiano et al., 2006). Moreover, the presence of mutant SOD1 in mitochondria can lead to an increase of the motor neurons sensitization to glutamatergic (over)-stimulation and therefore to excitotoxicity. Also, the inflammation and associated microglial activation, both hallmarks of ALS, may contribute to increasing the motor neurons’ susceptibility to excitotoxicity.

## The Old Man and the Alzheimer’s Disease

**Figure 4:**
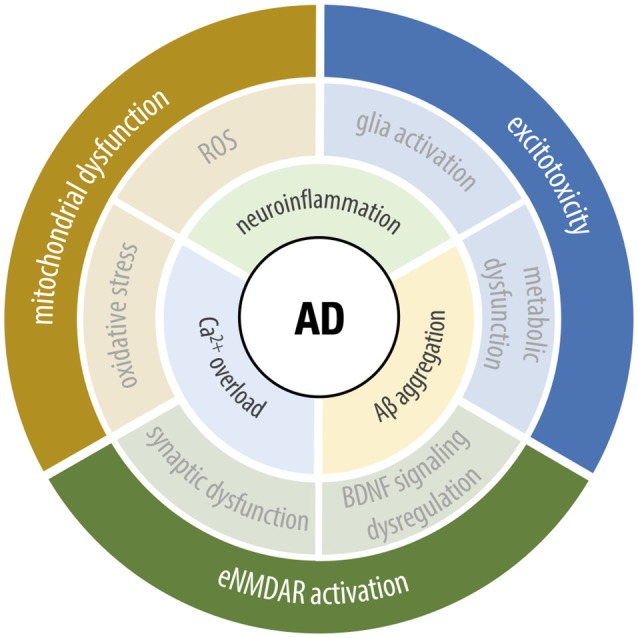
Underlying cellular mechanisms of Alzheimer’s disease (AD). The upstream key hallmarks of AD range from ionic dysfunction to the impairment of several cellular processes, including Ca^2+^-signaling dysregulation, abnormal Aβ production and aggregation and neuroinflammation. The crosstalk between the mentioned factors, leads to the pathological phenotype, involving mitochondrial dysfunction, excitotoxicity, and extrasynaptic NMDA receptor (eNMDAR) activation. As the symptomatic circle closes, the dysfunction of one leads to further activation of the others, altogether contributing to neurodegeneration and cognitive deficits, characteristics of the disease.

Alzheimer’s Disease (AD) is an age-dependent neurodegenerative disease, considered the most common form of dementia worldwide (Alzheimer’s Association, 2009). AD is firstly manifested by the loss of episodic memory and later loss of executive functions like language, attention, and reasoning (LaFerla et al., 2007). AD is commonly characterized by the presence of senile plaques, large extracellular aggregates of fibrillary amyloid-β peptide (Aβ), originated by the abnormal cleavage of amyloid precursor protein where the sequential cleavage of amyloid precursor protein by β- and γ-secretase produces the neurotoxic Aβ peptide (Scheuner et al., 1996; Ertekin-Taner, 2007). The presence of intracellular neurofibrillary tangles of hyperphosphorylated tau protein is also a characteristic of AD (Grundke-Iqbal et al., 1986; Ferrer, 2012). Many pieces of evidence suggest that Aβ_1–42_ oligomers are the most toxic Aβ species, exerting their pathological actions by disrupting glutamatergic transmission, mainly by acting on NMDAR and mGluR (Walsh et al., 2002). Aβ can also increase glutamate release from both neurons (Brito-Moreira et al., 2011) and astrocytes (Talantova et al., 2013), resulting in abnormally high extracellular glutamate levels capable of activating eNMDAR and, thus, leading to the activation of pro-death pathways and consequent excitotoxicity. Hence, the harmful effects of Aβ in AD may be mediated by the excessive activation of eNMDAR containing predominantly GluN2B subunits (Li et al., 2011). Also, it has been demonstrated that the presence of Aβ can induce a sustained calcium influx *via* NMDAR (Texidó et al., 2011), which can trigger a cascade of events leading to mitochondrial and synaptic dysfunction, excitotoxicity, production of ROS, and neuronal death (Ferreira et al., 2012), as depicted in Figure 4.

An imbalance between sNMDAR and eNMDAR activity was observed in brain samples from AD patients, which can contribute to the Aβ-triggered neurotoxicity observed in this disease. In parallel, it is also known that stimulation of eNMDAR plays a critical role in AD. Recent studies performed in rat acute hippocampal slices under Aβ exposure and in Aβ-injected mice support the idea that enhancement of sNMDAR activity, together with the inhibition of eNMDAR, has protective effects against Aβ-induced neurotoxicity (Huang et al., 2017). eNMDAR stimulation is also associated with the inactivation of extracellular signal-regulated kinase/mitogen-activated protein kinase signaling (Mulholland et al., 2008), which is crucial for memory consolidation and synaptic plasticity, suggesting that eNMDAR activation leads to the impairment of the molecular mechanisms underlying memory and learning processes (Schafe et al., 2000). In the presence of Aβ, the exacerbated calcium influx *via* eNMDAR is also associated with excessive activation of calpains, which are calcium-dependent proteases involved in multiple cellular functions, including proliferation, differentiation, and modulation of synaptic function (Wu and Lynch, 2006). The overactivation of calpains in AD is associated with functional changes in several proteins involved in neuronal transmission, such as BDNF. Previous reports have shown that Aβ impairs BDNF function in a calpain-dependent manner (Jerónimo-Santos et al., 2015), whereas inhibition of eNMDAR can prevent Aβ-triggered impairment of BDNF action in long-term synaptic potentiation (Tanqueiro et al., 2018).

Under physiological conditions, mitochondrial calcium signaling stimulates oxidative phosphorylation and ATP synthesis. In AD, as a consequence of the exacerbated calcium levels derived from eNMDAR activation, mitochondria form a non-specific pore in their internal membrane, termed mitochondrial permeability transition pore (Leung et al., 2008). With the pore opening, protons can cross this membrane, leading to the loss of mitochondrial membrane potential and dysregulation of pH gradient. Consequently, this affects ATP synthesis by oxidative phosphorylation, resulting in ionic and metabolic impairment, inevitably leading to cell death. Moreover, this decrease in mitochondrial bioenergetic capacity and consequent impairment of oxidative phosphorylation is associated with free radical production and subsequent oxidative damage. There are evidences of cyclophilin D (CypD), an integral part of the mitochondrial permeability transition pore (Leung et al., 2008), playing a critical role in Aβ-induced mitochondrial and synaptic injury (Du et al., 2008, 2011). Neuronal CypD is linked to Aβ-mediated ROS production, whereas CypD-deficient neurons appear to be resistant to Aβ-mediated inactivation of protein kinase A/CREB signaling, thus revealing that the absence of CypD can prevent Aβ-induced mitochondrial and synaptic dysfunction (Du et al., 2014). Additionally, mitochondrial dysfunction in AD includes respiration deficits, increased generation and accumulation of free radicals, and impaired energy metabolism (Hensley et al., 1994; Manczak et al., 2006; Yao et al., 2009; Calkins et al., 2011). Changes in mitochondrial dynamics, including decreased transport, increased fission, and, eventually, loss of mitochondrial function, are also observed in AD (Hensley et al., 1994; Manczak et al., 2006; Yao et al., 2009; Calkins et al., 2011).

Dysfunctional mitochondria are considered the main generator of ROS and consequent oxidative stress in AD, where oxidative stress can alter protein kinase A/CREB signaling, a signaling pathway necessary for neuronal survival (Wang et al., 2014). Indeed, several reports indicate that alterations in brain glucose metabolism in AD patients contribute to synaptic dysfunction and neuronal loss (Kennedy et al., 1995; Ishii et al., 1997). ROS, as well as reactive nitrogen species, are associated with the formation of protein carbonyls and 3-nitrotyrosine, which correlates with the level of protein oxidation within a cell. The determination of protein carbonyls levels and tyrosine nitration is considered viable measures of oxidative damage, where tyrosine nitration is one specific form of protein oxidation that is associated with AD (Castegna et al., 2003), which culminates in the loss of protein function, cellular dysfunction and, ultimately, cell death (Butterfield and Stadtman, 1997; Butterfield et al., 2006).

In AD, glial cell activation and consequent pro-inflammatory response is associated with an increased expression of NOS, an enzyme responsible for NO production. Indeed, inducible NOS, one of the NOS isoforms, has been found in activated astrocytes and microglia, suggesting a critical role of this enzyme in pathological conditions (Diaz et al., 2011). Also, inducible NOS has been described as the main culprit behind the NO increase to neurotoxic levels, whereas its inhibition was found to be a useful target for neuronal protection against Aβ-mediated toxicity (Diaz et al., 2011). Furthermore, a recent report found that, in adult rats injected with Aβ_25–35_, a neurotoxic Aβ fragment, there is an increase in inducible NOS expression and consequent upsurge of NO levels, which was prevented by the administration of a highly selective cannabinoid receptor 1 agonist, ACEA (Patricio-Martínez et al., 2019). Additionally, animals injected with ACEA and Aβ_25–35_ presented an improvement in learning and memory when compared with animals injected with Aβ_25–35_ alone (Patricio-Martínez et al., 2019), suggesting the involvement of the cannabinoid receptor 1 in neuroprotective mechanisms in AD.

Growing evidence has identified reactive astrocytes as a key player in glutamate-mediated excitotoxicity, since astrogliosis results in the loss of astrocytic physiological function and consequent impairment of neuronal synaptic transmission. This phenomenon is common in AD, where Aβ induces functional, morphological, and metabolic astrocytic dysfunction (Angelova and Abramov, 2014; Brawek and Garaschuk, 2014). Specifically, a downregulation of astrocytic glutamate transporters has been shown in both animal models and AD human brain samples, impairing glutamate uptake and causing excitatory overload in the synaptic cleft. Moreover, in astrocytic cell culture, Aβ reduces the expression of GLT-1 through a mechanism that involves the calcineurin/nuclear factor of activated T cells pathway (Abdul et al., 2009) and oxidative stress (Scimemi et al., 2013). This reduction of GLT-1 expression in astrocytes results in increased glutamate levels at the synapse, which, by acting on eNMDAR, contributes to the progression of AD pathology in the human brain (Simpson et al., 2010). Additionally, a study performed in postmortem human samples suggests that the preservation of GLT-1 expression in reactive astrocytes could act as a neuroprotective mechanism against AD neuropathological changes (Kobayashi et al., 2018).

Astrocytic glutamate uptake is not only essential for synaptic glutamate clearance, but also the activation of the astrocytic glycolysis cycle. The impaired glutamate uptake will then lead to glycolysis deficiency. In this scenario, neurons not only suffer the consequences of the excessive extracellular glutamate but also of metabolic insufficiency as the astrocytes fail to fulfill the necessary energetic needs. In the hippocampus, glycogen-derived lactate is responsible for memory formation and long-term synaptic potentiation (Newman et al., 2011). Inhibition of lactate production and neuronal delivery weakens synaptic plasticity and memory formation, by impairment of the Krebs cycle and cell signaling dysregulation (Barros, 2013; Schurr, 2014; Dienel, 2017). Indeed, when Long-Evans rats with induced astrocytic metabolic impairment were treated with downstream glycolytic metabolites, such as pyruvate or β-hydroxybutyrate, memory improvement and consolidation were observed (Descalzi et al., 2019). This may suggest that astrocytic metabolic impairment is responsible for memory loss and cognitive impairment, even in the premature stages of AD (Merlini et al., 2011).

Metabolic dysfunction is a very early sign of disease onset. Some studies suggest that glucose hypometabolism is a hallmark of AD that can be detected long before any significant Aβ plaques and neurofibrillary tangles (Mosconi et al., 2009; Chen and Zhong, 2013). As the first aggregates occur with concomitant astrocytic dysfunction, signaling for glycolysis starts to drop and leads to the onset of energetic failure, causing metabolic starvation and consequent excitotoxicity. So far, it has been difficult to determine the causal relationship between astrocytic glutamate uptake dysregulation and metabolic disturbance. Nevertheless, the contribution of the metabolic processes in the pathological excitotoxicity is undeniable.

The role of neuroinflammation in the chemically induced AD animal models has been proven significant for the neurodegenerative profile and assumed to be a crucial factor in disease progression (Liu and Hong, 2003; Reynolds et al., 2007; Taylor et al., 2013). In the early stages of AD, activated microglia express both neurodegenerative (M1) and neuroprotective (M2) phenotypes (Edwards et al., 2006). The M2 phenotype exhibits a great capacity for Aβ and tau protein aggregates clearance through phagocytosis. During disease progression, however, the M2 phenotype appears to undergo polarization into an M1 phenotype, resulting in the overproduction of neurotoxic factors. Indeed, in all stages of AD, significant aggregation of M1 microglia has been observed surrounding neurofibrillary tangles (Sheffield et al., 2000; Yao and Zu, 2020). M1 microglia not only release neurotoxic factors but have also been shown to downregulate or even block neuroprotective mechanisms involved in phagocytosis of Aβ (Yamamoto et al., 2008). This inhibition is mainly due to the impairment of the Aβ receptor complex, complement receptor 3, and insulin-degrading enzyme expression (Koenigsknecht-Talboo and Landreth, 2005). Among neurotoxic factors released by microglia, microglial glutamate release can stimulate excitotoxicity (Barger et al., 2007), while the release of pro-inflammatory cytokines such as interferon γ and TNFα impair the uptake and internal degradation of extracellular glutamate (Hu et al., 2000; Yamamoto et al., 2008). In AD, reactive astrocytes are also able to release pro-inflammatory cytokines, such as, TNFα and interleukin 1-β, which, while not directly cytotoxic, can impair astrocytic glutamate uptake and to enhance NMDAR-induced calcium release, contributing to AD progression. Moreover, it is known that inhibitors of these inflammatory mediators attenuate both synaptic and cognitive deficits in Aβ-treated mice, suggesting that these inflammatory responses mediated by astrocytes have negative effects upon synaptic plasticity mechanisms (Ralay Ranaivo et al., 2006).

Though not often discussed, the cystine-glutamate (Xc-) antiporter system is essential for cysteine homeostasis and considered only a secondary mechanism in the glutamate release and uptake. However, its contribution to pathological states such as glutamate excitotoxicity should not be ignored. The Xc- antiporter system is highly expressed in microglia and astrocytes. It is a sodium-independent anionic amino acid transporter with high specificity for negative species of cysteine and glutamate (Sato et al., 2002). It is also electroneutral, instead of electrogenic as most common EAAT systems (Lo et al., 2008). This antiporter is a heterodimer (Lutgen et al., 2014), composed of heavy 4F2 chain and a specific light chain, named xCT, linked by a disulfide bridge (Lewerenz et al., 2012). It uptakes cystine in exchange for releasing glutamate in the molar ratio 1:1 (Sato et al., 1999). Although less discussed, the Xc- system significantly increases extracellular and, more specifically, synaptic levels of glutamate, releasing up to 60% of all extracellular glutamate in the striatum (Baker et al., 2002). This antiporter can release up to 0.6 μM/s of glutamate into the extracellular space (Warr et al., 1999), which is then uptaken by GLT-1 (Baker et al., 2002; Bridges et al., 2012). Depending on the influx and overall capacity to uptake glutamate, this release can either activate or desensitize the NMDAR (Warr et al., 1999). The cystine-glutamate antiporter was reported to exhibit neuroprotective effects in neurodegenerative diseases, as it is linked to the production of glutathione and the rise of antioxidant cellular capacity (Bridges et al., 2012). This protective effect is observed in AD models, where the Xc- system uptakes cystine, showing great potential at lowering Aβ-induced toxicity, oxidative stress, and later apoptosis. However, when activated either by oxidative stress, TNF-α, or amyloid precursor protein, this antiporter can release cytotoxic amounts of glutamate (Barger and Basile, 2001; Sato et al., 2001). Not only is this antiporter involved in the release of glutamate, contributing to excitotoxicity, but it has also been shown to block microglial neuroprotective functions in AD. Indeed, activated microglia in both AD patients and in AD mouse models show an increased expression of this antiporter and, specifically, the xCT protein (Bridges et al., 2012). In mice, transgenic depletion of the *Slca11* gene (xCT^–/–^ mice) impaired microglial polarization into the neurotoxic phenotype and reduced up to 70% pathological glutamate release in cultured microglial cells (Mesci et al., 2015). Upon blockade of either NMDAR or the Xc- system, microglia act in a neuroprotective way when exposed to Aβ and attenuate neuronal death. However, when neurons were cultured with microglia depleted of lipid-associated apolipoprotein E expression, the neuroprotective phenotype was lost (Qin et al., 2006), implying that the neuroprotective phenotype of microglia in AD is related to apolipoprotein E. In neuron-microglia co-cultures, the activation of the Xc- system by the Aβ deposits not only exacerbates excitotoxicity, but also lowers the neuronal threshold for glutamate toxicity, allowing non-toxic concentrations of glutamate to exert excitotoxic actions (Qin et al., 2006). Although very sparse in current literature, the neuroprotective role of the Xc- system *in vivo* showed similar effects, where the activation of microglial cells by Aβ can cause both phenotypes, the neurotoxic being mostly related to glutamate release and increased neuronal susceptibility to excitotoxicity, and the neuroprotective related to expression of apolipoprotein E and glutathione (Bannai and Tateishi, 1986; Qin et al., 2006; Shih et al., 2006).

As stated before, another AD hallmark is hypoglycemia. Removal of glucose in mixed astrocyte-neuron cell cultures leads to a glycemic neuronal injury and death (Thorn et al., 2015). However, the neuronal death observed in glucose deprivation conditions is not connected to energy failure but most likely tied to glutamate accumulation in the synaptic cleft (Jackman et al., 2012). Astrocytes express significant levels of the cystine-glutamate antiporter and are also the main metabolic factories and energy suppliers for neurons. As these processes are tightly linked, the dysfunction of one is likely to mediate the function of the other system, for the benefit or damage. Upon glucose deprivation in cultured astrocytes, xCT protein expression rose in a time-dependent matter (Thorn et al., 2015).

In the triple transgenic AD mouse model (3xTg-AD), astrocytic glutamine synthetase expression was found to be decreased in the hippocampus, suggesting a critical role of astrocytes in AD-related disruption of glutamate homeostasis, which may affect the efficiency of glutamatergic transmission, contributing to the cognitive deficits of the disease (Olabarria et al., 2011). Also, it is known that glutamine synthetase activity is sensitive to oxidation and may be impaired by oxidative stress in AD (Smith et al., 1991; Hensley et al., 1994, 1995). In the 3xTg-AD mouse model, Aβ near to astrocytes leads to the extrasynaptic release of glutamate and consequent eNMDAR activation (Talantova et al., 2013), which contributes to synaptic dysfunction and neuronal death. Furthermore, there are also evidences that eNMDAR activity can trigger the generation of toxic Aβ (Talantova et al., 2013), which suggests a key role of eNMDAR and astrocytic glutamate release in mediating Aβ neurotoxicity.

As mentioned before, astrocytes are responsible for the supply of D-serine to neurons (Henneberger et al., 2010). Indeed, recent evidences reveal that serine racemase, the enzyme responsible for D-serine synthesis is strongly expressed in reactive astrocytes in both human AD samples and AD rat models (Balu et al., 2019). Moreover, changes in intracellular signaling cascades, consistent with excitotoxicity and decreased neuronal survival were found (Balu et al., 2019). Hence, these findings support a model where D-serine released from reactive astrocytes could bind to extrasynaptic GluN2B-containing NMDAR, triggering the activation of excitotoxic signaling pathways and consequent neuronal damage and death. It has however been shown that, in AD-like conditions, the selective blockade of eNMDAR together with the administration of a D-serine-like NMDAR co-agonist significantly improved spatial and related forms of learning and memory (Huang et al., 2017). Moreover, there are evidences that blocking eNMDAR provides neuroprotection against Aβ-triggered deficits, together with the enhancement of sNMDAR activity (Huang et al., 2017). In conclusion, while it is known that D-serine is mainly a co-agonist of sNMDAR, the role of D-serine as a mediator of neuronal excitotoxicity cannot be excluded.

**Figure 5:**
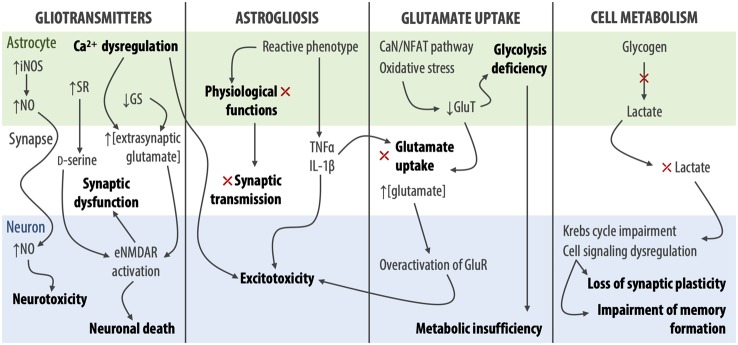
Aβ-induced astrocytic dysfunctions leading to excitotoxicity and AD pathogenesis. iNOS, inducible nitric oxide synthase; NO, nitric oxide; SR, serine racemase; GS, glutamine synthetase; CaN/NFAT—calcineurin/nuclear factor of activated T cells; GluT, glutamate transporters; GluR, glutamate receptors.

In line with what was described above, many studies support the active involvement of glial cells, in particular astrocytes, in early stages of AD pathogenesis, where Aβ can dysregulate astrocytic calcium signaling both *in vitro* and *in vivo* (Haughey and Mattson, 2003; Kuchibhotla et al., 2009), which can result in the excessive release of glutamate and other gliotransmitters, enhancing extrasynaptic glutamatergic signaling and consequent excitotoxicity in AD, as summarized in Figure 5.

## The Phantom of the Brain: Epilepsy

**Figure 6:**
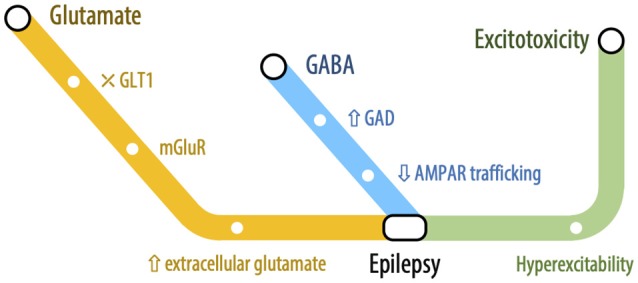
The connecting railway between Epilepsy and Excitotoxicity: Glutamatergic and GABAergic mechanisms in epilepsy-related excitotoxicity. Schematic representation of alterations in excitatory and inhibitory neurotransmission and their relationship with epilepsy-excitotoxicity dynamic. Both alterations in glutamate- and GABA-mediated neurotransmission have been reported to play a role in epileptogenesis. Increased glutamate levels are a key-feature of temporal lobe epilepsy, resulting from an impairment in GLT-1, which are compensated by increased GAD. The excessive and hypersynchronous neuronal firing promotes network hyperexcitability, culminating in glutamate-mediated excitotoxicity.

Epilepsy is one of the most common neurological disorders, estimated to affect more than 70 million people worldwide (Singh and Trevick, 2016). Epilepsy can be regarded as a family of disorders which comprise several diseases and conditions and can be defined by: (i) the unprovoked occurrence of at least two seizures more than 24 h apart; (ii) one unprovoked seizure and further seizure probability of at least 60%; or (iii) diagnosis of an epilepsy syndrome. There are multiple types of seizures, which are characterized in accordance with their onset, physical manifestation, and level of consciousness, differing in the corresponding pathophysiological mechanisms (Fisher et al., 2017). Nevertheless, all epileptic seizures involve persistent changes in synaptic events such as neurotransmitter release, and receptor and transporter activity, and are underpinned by an abnormal excessive or synchronous neuronal firing activity, typically resulting from an imbalance between excitatory and inhibitory processes mediated by the neurotransmitters glutamate and GABA, respectively. Excessive neuronal firing is accompanied by increased extracellular levels of glutamate, leading to excitotoxicity (Soukupová et al., 2014), one of the primary sources of neuronal damage in epilepsy. The processes by which excitotoxicity results in neuronal damage and/or death have previously been described and, in this section, we will focus on the underlying pathophysiological mechanisms of different types of epilepsy and how they correlate with excitotoxicity. The main findings discussed here are summarized in Figure 6.

### Temporal Side Story: Temporal Lobe Epilepsy

Temporal lobe epilepsy (TLE) is the most common form of epilepsy, affecting up to 40 million people worldwide (Hubbard et al., 2016; Peterson and Binder, 2019). TLE is characterized by the occurrence of focal seizures that may impair individuals and is often associated with treatment resistance, where approximately 30% of patients taking antiepileptic drugs present drug resistance that prevents them from living seizure-free (Sarac et al., 2009; Hubbard et al., 2016; Peterson and Binder, 2019).

The increase in local excitation and cytotoxicity in the epileptogenic focus of TLE is linked to an increase in extracellular glutamate levels (Proper et al., 2002; Sarac et al., 2009; Albrecht and Zielinska, 2017). Changes in glutamate levels have been reported in the hippocampus of epileptic patients before, during, and after seizures, which may occur due to increased glutamate release or its impaired reuptake (Proper et al., 2002; Hoogland et al., 2004; Hubbard et al., 2016).

As mentioned before, glutamate uptake is a major determinant of extracellular glutamate levels and evidence shows that the alteration of glutamate concentrations in epilepsy may result from aberrant EAAT expression or function (Crino et al., 2002; Proper et al., 2002). Indeed, an increase in EAAT3 expression in the hippocampus of both patients and animal models with TLE has been observed (Crino et al., 2002). This enhanced expression may lead to the increased glutamate release during seizures by increasing intracellular glutamate, which will be stored in vesicles and synaptically released by the classical calcium-dependent pathway (During and Spencer, 1993; Crino et al., 2002). Another mechanism by which increased glutamate release may occur is EAAT3 reversal due to the alteration of cellular excitability promoted during epileptogenesis (Rossi et al., 2000). However, the increase in EAAT3 expression may be a compensatory mechanism, leading to the improvement of GABAergic inhibitory transmission, as shown in the granular cells of the dentate gyrus of TLE animal models, which had increased glutamate decarboxylase expression and GABA release (Sloviter et al., 1996; Crino et al., 2002).

Additionally, reactive astrocytes with morphological and functional alterations may be important in epileptogenesis since they contribute to the increase of neuronal excitability. In TLE, this astrocyte-induced excitotoxicity occurs by dysregulation of glutamate astrocytic transporters (EAAT1 or GLAST and EAAT2 or GLT-1), which are responsible for maintaining extracellular glutamate homeostasis (Hubbard et al., 2016; Peterson and Binder, 2019). Different studies have shown that there is a reduction in EAAT1 and EAAT2 expression in the sclerotic hippocampus of TLE patients at RNA or protein levels (Proper et al., 2002; Rakhade and Loeb, 2008). Furthermore, modifications in GLT-1 function and expression contribute to TLE hyperexcitability and seizure generation (Hubbard et al., 2016; Peterson and Binder, 2019). Different studies have already shown that knockout animal models for GLT-1 exhibit severe spontaneous seizures that can lead to early postnatal death (Tanaka et al., 1997; Hubbard et al., 2016; Peterson and Binder, 2019), and that GLT-1 overexpression may generate pilocarpine-induced epileptogenesis resistant mice (Kong et al., 2012; Sha et al., 2017). In fact, in the intrahippocampal kainic acid mouse model of TLE, a down-regulation of hippocampal astrocytic GLT-1 has been reported, coinciding with the increased excitability (Peterson and Binder, 2019). Also, there seems to be a temporal regulation of GLT-1 expression during epileptic seizures, with an increase in GLT-1 levels 1–4 days after seizure and a reduction 4–7 days after a seizure (Hubbard et al., 2016; Peterson and Binder, 2019). This difference in GLT-1 expression after epileptic seizures can arise from changes in the membrane anchor and trafficking system, since, upon glutamate release, GLT-1 can be trafficked to synaptic and non-synaptic regions to ensure glutamate clearance (Murphy-Royal et al., 2015). Different drugs can ameliorate epilepsy intensity by targeting GLT-1. Among these drugs, antibiotics such as β-lactam and ceftriaxone show antiepileptic effects and can increase GLT-1 levels (Rothstein et al., 2005; Zeng et al., 2010), and, in a pilocarpine-induced TLE model, a GLT-1 activator was able to promote an increase in GLT-1 expression and reduction of spontaneous seizures frequency by 50% (Kong et al., 2014; Sha et al., 2017). However, GLT-1 internalization and degradation are still observed, suggesting that these are the causes of GLT-1 deficiency and the limiting factors for the use of these drugs in the treatment of TLE (Susarla and Robinson, 2008). Thus, some studies have shown that besides increasing GLT-1 expression, it would be necessary to use inhibitors of proteins responsible for its degradation. Studies have been performed with a Hsp90B inhibitor (17AAG), which is one of the proteins that recruit GLT-1 for degradation (Whitesell and Lindquist, 2005). Not only was this drug able to increase GLT-1 expression, it also maintained GLT-1 protein levels more effectively than ceftriaxone and exhibited a remarkable seizure suppression effect in the TLE model (Sha et al., 2017).

Besides glutamate homeostasis, astrocytes can also modulate water flow and potassium homeostasis in the extracellular space (Devinsky et al., 2013). Potassium concentration is regulated by astrocytes through its uptake by the sodium/potassium ATPase, sodium/potassium/chloride cotransporters, and input rectifier channels for potassium (Kir 4.1; Ransom et al., 2000; D’Ambrosio et al., 2002; Kofuji and Newman, 2004; Nikolic et al., 2019). Increased potassium concentration in the extracellular space can generate sustained neuronal depolarization and neuronal hyperexcitability (Walz, 2000; Devinsky et al., 2013; Nikolic et al., 2019), and evidence in the literature suggests an association between the uncontrolled increase of extracellular potassium levels and epilepsy, both in humans and in animal models (Steinhäuser et al., 2016).

In recent years, studies have shown an association between the glial water channel aquaporin-4 (AQP4) and Kir 4.1, forming an astrocytic protein complex responsible for removing potassium from the extracellular space, being an important mechanism in establishing a hyperpolarized neuronal membrane potential which may play an important role in epilepsy (Aronica et al., 2000; Das et al., 2012; Devinsky et al., 2013). Indeed, reduced expression of Kir 4.1 has been described in the hippocampus of patients with TLE (Das et al., 2012), where astrocytes showed deficits in potassium and glutamate uptake similar to those found in Kir 4.1 knockout animal models (Djukic et al., 2007; Chever et al., 2010; Nikolic et al., 2019). Furthermore, AQP4 knockout animals are also more susceptible to seizures and epilepsy (Dudek and Rogawski, 2005; Devinsky et al., 2013) and, in kainate-induced epileptic animal models, a reduction in AQP4 expression was also reported (Lee et al., 2012; Devinsky et al., 2013). Similarly, patients with TLE who have hippocampal sclerosis show a decrease or loss of AQP4 expression, suggesting the involvement of these channels in early epileptogenesis (Amiry-Moghaddam et al., 2003; Eid et al., 2005; Seifert et al., 2010). Taken together, these findings show that, in addition to the glutamate transporters, the control of potassium homeostasis and water flow is involved in neuronal excitability and TLE, even though further studies are needed to better elucidate their underlying mechanisms for controlling neuronal excitability.

Not only is ion homeostasis, such as sodium and potassium, altered in various forms of epilepsy, but cell metabolism has also been shown to be deeply affected in the epileptic brain. For instance, it is well known that there is a high energy demand during seizures, accompanied by high glucose consumption by neural networks. However, cerebral glucose hypometabolism is a feature of interictal phases in different forms of epilepsy, in regions that encompass but are generally larger than the region of seizure onset (Goffin et al., 2008; Pittau et al., 2014). The exact mechanisms underpinning such alterations are not clear, but mitochondrial dysfunction, linked with deficient glycolysis, remains a plausible hypothesis. Indeed, glutamate is known to promote astrocytic glycolysis (Bittner et al., 2011; Yan et al., 2017), a process in which glucose is converted into lactate. In epilepsy, where extracellular glutamate levels are increased, this process is most likely heightened when in comparison with control conditions. Thus, this results in a rapid depletion of glucose and a quick increase in lactate levels, indicating that, following seizure activity, in which there is a high consumption of glucose, neurons likely rely mostly on astrocyte-derived lactate. The lactate surplus is released into the extracellular space, creating a gradient between astrocytes and neurons (Mächler et al., 2016). Accompanying these alterations, the expression of astroglial monocarboxylate transporters, which lead to an efflux of monocarboxylates in astrocytes, has been reported to be increased in animal models of TLE (Liu et al., 2014), further suggesting that this astroglial-neuronal metabolic coupling pathway is affected in epilepsy. This pathway is vital for several brain functions, including for long-term memory formation (Suzuki et al., 2011), which might indicate a compromise of several neural functions, as a consequence of disrupted energy homeostasis. Due to the accumulating evidence of metabolic disruption in epilepsy, treatment strategies targetting cell metabolism have been gaining more attention, including the revival of the ketogenic diet (Walczyk and Wick, 2017). Pyruvate has been proposed as a possible treatment for epilepsy on account of being an anaplerotic mitochondrial fuel and because of its protective features, including acting as a potent ROS scavenger and normalizing cytosolic redox state. This molecule also facilitates brain-to-blood glutamate flux, preventing neuronal excitability (Popova et al., 2017). Finally, since blood glutamate can be converted into 2-ketoglutarate in the presence of pyruvate, mediated by glutamate-pyruvate transaminase (Gottlieb et al., 2003), treatment with pyruvate can be a protective measure against excitotoxicity (Carvalho et al., 2011).

In addition to alterations in cell metabolism in epilepsy, which include the hypermetabolism and hypometabolism during ictal and interictal phases, respectively, intracellular ATP levels are also affected. Indeed, depletion of intracellular ATP has been reported in seizures (Wasterlain et al., 2010), and considering that the major source of ATP in the mitochondria, the possibility of mitochondrial dysfunction in epilepsy has gained considerable attention. While it is known that mitochondrial dysfunction is involved in seizure-related neuronal death (Kovac et al., 2013), it has also been associated with increased ROS and NO production during seizures (Frantseva et al., 2000; Jarrett et al., 2008), which is related with the activation of glutamate receptors and depolarization of the mitochondrial membrane. As the major sources of ATP are glycolysis and mitochondrial respiration, alterations in the rate of these processes in epilepsy will also impact ATP production which, reciprocally, influences mitochondrial activity. Mitochondrial dysfunction has been profoundly linked with neuronal damage and death (Rose et al., 2017), and this organelle is particularly relevant for calcium homeostasis. Mitochondria serve as a key regulator of cytosolic calcium, *via* the mitochondrial calcium uniporter and, in its turn, the influx of calcium into the mitochondria can activate the respiratory chain as well as promote the production of ATP. During seizure activity, in which neurons are exposed to high concentrations of glutamate, the subsequent activation of NMDAR induces calcium oscillations and a subsequent depolarization of the mitochondrial membrane (Kovac et al., 2012), which can be a trigger for neuronal death (Vergun et al., 1999). In rats where epileptic status was induced by L-allylglycine and bicuculline, the influx of calcium into neurons led to mitochondrial calcium overload, resulting in cell death (Griffiths et al., 1983; De Stefani et al., 2011). This issue has been overcome in the pilocarpine model of epilepsy by the inhibition of the mitochondrial calcium uniporter system, which decreased neuronal death, suggesting this system plays a role in seizure activity (Wang et al., 2015). Also, increasing ATP production by providing a substrate such as a pyruvate has been demonstrated to prevent neuronal death (Kovac et al., 2012). It should be noted that not only mitochondrial alterations can be observed but also in the endoplasmic reticulum, another intracellular pool of calcium. Indeed, debilitation of endoplasmic reticulum calcium with thapsigargin results in decreased neuronal excitation triggered by bicuculline in cultures (Sokal et al., 2000) as well as in slices, where (RS)-3,5-dihydroxyphenylglycine and pilocarpine-induced ictal discharges were shown to be dependent on the endoplasmic reticulum calcium pool (Rutecki et al., 2002). Although very few studies have focused on the contribution of endoplasmic reticulum calcium stores toward seizure activity, it has been demonstrated that depleting intracellular endoplasmic reticulum calcium stores can counteract neuronal excitability (Sokal et al., 2000).

Another major consequence of intracellular calcium accumulation and mitochondrial dysregulation is the exacerbated production of ROS, which is an essential mechanism in excitotoxicity-induced neuronal injury (Patel et al., 1996). Indeed, oxidative stress has been implicated in both immediate and later excitotoxic neuronal damage and increased ROS production has been reported in the ictal and interictal phases of epilepsy (Liang et al., 2000). Furthermore, this increase is also known to inhibit mitochondrial complex 1 activity and decrease mitochondrial membrane potential (Ryan et al., 2012; Rowley et al., 2015).

Additionally, calcium influx also leads to NOS activation and consequent NO overproduction, which is known to be increased in the cerebral cortex of rodents and epilepsy patients (Mülsch et al., 1994; González-Hernández et al., 2000) and has been suggested to contribute to seizure-induced neuronal death. It has also been demonstrated that NO-mediated activation of the type 1 ryanodine receptor is crucial for seizure-induced neuronal death and that antagonists of this receptor have a neuroprotective effect, emerging as suitable candidates for ameliorating conditions following seizures (Mikami et al., 2016). Supporting this fact, it has been described that non-selectively blocking NOS can potentiate rubidium chloride anti-convulsive effects (Rahimi et al., 2019). Thus, given the onset of metabolic and mitochondrial alterations as a consequence of excitotoxicity in seizure activity and their relevance for neuronal damage, there has been a growing interest in the use of antioxidants and other neuroprotective drugs that prevent neuronal damage following seizures (Petkova et al., 2014; Mishra et al., 2015; Williams et al., 2015; Pauletti et al., 2019).

## Curtain Call: Conclusions

Excitotoxicity relies on multiple pathways that are involved in several regulatory cell mechanisms. Studies reviewed here suggest that, although the glutamatergic system is essential for brain functioning, its dysregulation is implicated as a key step in the pathophysiology of neuronal impairment, which is closely associated with excitotoxicity. This dysregulation may occur at the receptor, transporter, or metabolic levels, leading to different types of cellular responses that ultimately culminate in oxidative stress and neuronal death. Several neurodegenerative diseases display excitotoxic events or characteristics, and possibly this chronic mild excitotoxicity contributes, among other factors, to the neuronal death observed in these pathophysiological conditions. Thus, it is urgent to discover new therapeutic targets that counteract the excitotoxicity mediated by glutamatergic system, with a positive impact on neurological diseases such as ALS, AD, and TLE.

## Author Contributions

AA-M, JG, CP, OS, JG-R, NR, SP, TM, RM, and FR wrote the manuscript. AA-M designed the illustrations. AS and VC revised the manuscript. SV wrote, designed, and revised the manuscript.

## Conflict of Interest

The authors declare that the research was conducted in the absence of any commercial or financial relationships that could be construed as a potential conflict of interest.

## Abbreviations

AD: Alzheimer’s Disease
ALS: amyotrophic lateral sclerosis
AMPAR: α-amino-3-hydroxy-5-methylisoxazole-4-propionate receptors
AQP4: aquaporin 4
Aβ: amyloid-β peptide
BDNF: brain-derived neurotrophic factor
CREB: cAMP-regulatory element binding protein
CypD: cyclophilin D
EAAT: excitatory amino acid transporters
eNMDAR: extrasynaptic NMDAR
GABA: γ-aminobutyric acid
GABAR: GABA receptors
iGluR: ionotropic glutamate receptors
KAR: kainate receptors
Kir4.1: input rectifier channels for potassium
mGluR: metabotropic glutamate receptors
NMDAR: N-methyl-D-aspartic acid receptors
NO: nitric oxide
NOS: NO synthase
ROS: reactive oxygen species
sNMDAR: synaptic NMDAR
SOD: superoxide dismutase
TLE: temporal lobe epilepsy
TNF-α: tumor necrosis factor-α.
